# Comparative Study on the Response of Hyssop (*Hyssopus officinalis* L.), Salvia (*Salvia officinalis* L.), and Oregano (*Origanum vulgare* L.) to Drought Stress Under Foliar Application of Selenium

**DOI:** 10.3390/plants13212986

**Published:** 2024-10-25

**Authors:** Liubov Skrypnik, Pavel Maslennikov, Maria Antipina, Dmitriy Katserov, Pavel Feduraev

**Affiliations:** 1 Laboratory of Natural Antioxidants, Research and Education Center “Industrial Biotechnologies”, Immanuel Kant Baltic Federal University, 236040 Kaliningrad, Russia; 2 Scientific and Educational Cluster MEDBIO, Immanuel Kant Baltic Federal University, 236040 Kaliningrad, Russiapfeduraev@kantiana.ru (P.F.)

**Keywords:** abiotic stress, water-deficit stress, aromatic plants, reactive oxygen species, ascorbate–glutathione cycle, osmolyte, photosynthesis, irrigation

## Abstract

Drought is one of the most important abiotic factors limiting plant productivity. Although the aromatic plants of the Lamiaceae family often grow in arid regions, drought tolerance varies greatly among the different species of this family. The effect of induced drought stress can be reduced by the application of selenium. The current study aims to compare the growth and biochemical responses of three species of the Lamiaceae family (hyssop, salvia, and oregano) to drought stress and the possibility of reducing the effect of stress in these plants by foliar treatment with selenium. Drought stress reduced the fresh and dry biomass of hyssop (by 35% and 15%), salvia (by 45% and 41%), and oregano (by 51% and 32%). Se treatment did not affect the growth of plants under drought stress, but it improved relative water content in hyssop and salvia under moderate drought conditions. A reduction in the content of chlorophyll *a* and chlorophyll *b* (in hyssop and salvia). In addition, an increase in the content of hydrogen peroxide (in oregano and salvia), malondialdehyde, and proline in plants cultivated under drought conditions was observed. Se treatment led to reduced levels of hydrogen peroxide and malondialdehyde, along with an increase in chlorophyll *a* content (in hyssop and oregano) and proline content. The response of the antioxidant system depended on the plant species. Hyssop exhibited a significant increase in glutathione peroxidase, superoxide dismutase, and peroxidase activities. Oregano showed enhanced catalase activity. Salvia experienced a sharp increase in ascorbic acid content. Se treatment stimulated the accumulation of phenolic compounds and increased glutathione peroxidase activity in all studied species.

## 1. Introduction

Drought is one of the most critical limiting factors, significantly affecting plant growth, development, and productivity, thus impeding sustainable agriculture worldwide [1]. As a natural climatic phenomenon, drought occurs in almost all climatic zones, with variations in frequency, intensity, and duration [2]. Given the anticipated climate changes, it is evident that the frequency and duration of droughts will increase [3], positioning this as one of the major challenges of the 21st century and emphasizing the importance of this study.

The Lamiaceae family comprises over 6000 species of herbs and shrubs, which are globally distributed and have significant practical applications in pharmaceutical, food, and cosmetic industries [4]. This family includes plant species such as mint, thyme, rosemary, basil, lavender, oregano, salvia, and hyssop. Hyssop (*Hyssopus officinalis* L.) is a notable medicinal plant and a typical xerophyte, well adapted to drought and low-input conditions [5]. Salvia (*Salvia officinalis* L.) is a perennial woody shrub native to the Mediterranean region but is currently cultivated all over the world [6]. While various *Salvia* species exhibit relative drought tolerance, *S. officinalis* is particularly sensitive to water stress [7]. Oregano (*Origanum vulgare* L.), a perennial herbaceous species also originating from the Mediterranean, not only serves as a consumable plant but also contributes to sustainable agroecosystems and the reclamation of cultivated land [8]. Under water stress, oregano experiences a reduction in fresh weight, dry weight, relative water content (RWC), and total chlorophyll [9]. In this study, we selected three species from the Lamiaceae family, distinguished by their resistance to drought stress based on the literature data.

Plant responses to stress factors, including drought, are highly complex and occur at multiple levels, ranging from molecular changes to structural alterations in various parts of the plant. One of the most sensitive indicators of plants’ response to stress is the redox balance, which refers to the equilibrium between the production of reactive oxygen species (ROS) and the components of the antioxidant defense system [10]. Typically, exposure to abiotic stress factors such as salinity, drought, heavy metals, and metalloids triggers increased ROS production, along with changes in the activity of antioxidant enzymes and levels of non-enzymatic antioxidants [11,12,13]. Drought stress activates various signaling pathways, leading to changes in gene expression, which results in the production of functional compounds such as proline, glycine betaine, soluble sugars, late embryogenesis-abundant proteins, and aquaporins. These compounds are involved in plant metabolism and contribute to stress tolerance. Plants utilize two main signaling pathways—abscisic acid (ABA)-dependent and ABA-independent—to detect and respond to drought stress [14]. Regulatory gene products such as calcium-dependent protein kinases, mitogen-activated protein kinases, HD-ZIP/bZIP, AP2/ERF, NAC, MYB, and WRKY play key roles in regulating signal transduction pathways or acting as transcription factors, allowing plants to adapt to arid conditions by altering their morphology and physiology [15].

Although selenium (Se) has not yet been officially recognized as an essential microelement for plants, its significance has been demonstrated in numerous studies. A growing body of experimental evidence shows that selenium can enhance plant resistance to various abiotic stressors [16,17,18,19]. Notably, selenium treatment has been shown to improve the drought resistance of several plant species [20,21,22,23,24]. The protective effects of selenium are primarily linked to the activation of plants’ antioxidant system, particularly enzymes such as glutathione peroxidase (GPX), ascorbate peroxidase (APX), and catalase (CAT). Additionally, selenium helps regulate water balance, supports the photosynthetic apparatus, and stimulates the biosynthesis of osmoprotectants and secondary metabolites [21,25,26].

Due to the significant economic value of various Lamiaceae species, many studies have focused on the effects of drought stress on their growth and phytochemical composition, particularly on essential oils and phenolic compounds [27]. However, the data on selenium’s impact on the drought resistance of Lamiaceae species are limited and often contradictory. The goal of the present study was to verify the hypothesis according to which the difference in drought tolerance demonstrated for different Lamiaceae species is associated with species-specific reactions of the plant antioxidant system and the protective effect of selenium in plants under stress conditions determined by the regulation of cell redox metabolism. To test this hypothesis (1), a comparative analysis of the responses of three Lamiaceae species—hyssop, salvia, and oregano—to varying degrees of drought stress by examining changes in growth, photosynthetic pigment content, oxidative stress intensity, and antioxidant system activity, and (2) an assessment of the potential of foliar selenium treatment on the drought resistance of these species through its effect on the antioxidant system were conducted. The findings from this study will provide deeper insights into the mechanisms driving Lamiaceae plants’ response to drought stress and the role of selenium in regulating their stress resistance.

## 2. Results

### 2.1. Plant Growth Characteristics and Se Accumulation

Drought had a pronounced impact on the growth of hyssop, salvia, and oregano. The study revealed a significant reduction in shoot height (by 30%), fresh weight (by 35 to 50%), and dry weight (by 15 to 40%), depending on the species (Table 1). However, selenium treatment did not result in notable changes in growth indicators for any of the three species, regardless of the drought severity.

The RWC in hyssop, salvia, and oregano decreased significantly as drought stress intensified (Table 1). Selenium treatment, however, improved RWC in hyssop and salvia under moderate drought conditions. In oregano, Se did not affect RWC under moderate and severe drought stress, but significantly reduced it under control conditions.

The foliar application of exogenous selenium led to an increase in selenium concentration in the plant shoots (Table 2), with hyssop showing the highest selenium accumulation. The selenium concentration in hyssop shoots reached around 8–9 μg g^−1^, which was 2.3–3.5 times higher than in salvia and oregano. A three-way analysis of variance showed that the level of drought stress and the interaction between drought and selenium treatment did not influence selenium concentration in the plants (Supplementary Materials Table S1A).

### 2.2. Photosynthetic Pigments

Cultivating plants under drought conditions resulted in a reduction in chlorophyll *a* content (Table 3). At the highest drought stress level, the chlorophyll *a* content decreased by 1.6 times in hyssop, 1.4 times in salvia, and 1.3 times in oregano. However, treatment with exogenous selenium under drought conditions led to an increase in chlorophyll *a* content in hyssop and salvia (under moderate drought), as well as in oregano (under moderate and severe drought). Interestingly, in salvia under severe drought, selenium treatment resulted in a lower chlorophyll *a* content compared to untreated plants. 

Drought conditions also caused a reduction in chlorophyll *b* content in hyssop and salvia (Table 3). In contrast, the chlorophyll *b* content in oregano was unaffected by water availability or selenium treatment. Selenium treatment of hyssop cultivated under drought stress increased chlorophyll *b* content by 1.2 times compared to untreated plants. In salvia, selenium treatment led to an increase in chlorophyll *b* content under moderate drought, but a decrease under severe drought conditions.

According to the results of the 3-way analysis of variance, the chlorophyll *a*/chlorophyll *b* ratio was influenced by both plant species and the interaction between species and drought conditions (Supplementary Materials Table S1B). The most notable differences in this ratio were observed in oregano, where it decreased by 1.3 to 1.5 times under drought compared to control plants. Selenium treatment, however, significantly increased the chlorophyll *a*/chlorophyll *b* ratio in oregano under drought conditions.

The total chlorophyll content decreased in hyssop and salvia under drought conditions compared to the control, while no significant changes were observed in oregano (Table 3). Selenium treatment also had no effect on the total chlorophyll content in oregano. Notably, the effect of selenium on the total chlorophyll content in hyssop and salvia under drought followed the same pattern as the changes observed in chlorophyll *a* and chlorophyll *b* levels.

The effect of drought on carotenoid content was significant only in oregano (Table 3), where the highest carotenoid levels were recorded under maximum stress conditions. Selenium treatment did not significantly affect carotenoid content in any of the studied plant species.

### 2.3. Oxidative Stress Parameters 

**Figure 1:**
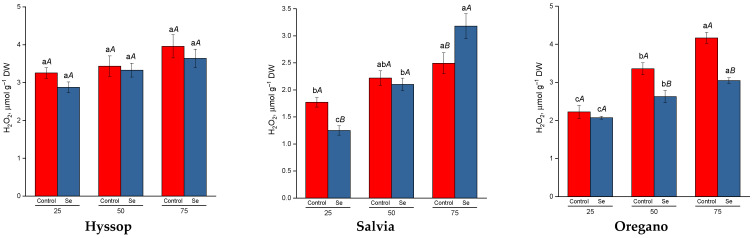
Effect of selenium on hydrogen peroxide content (H_2_O_2_) under drought stress. Different lower-case letters indicate significant differences between plants by drought level for control, Se-treatment, and each species separately, and upper-case letters indicate significant differences between control and Se-treatment at *p* ≤ 0.05 based on post hoc Tukey’s test (*n* = 4).

Cultivation of salvia and oregano under drought conditions resulted in a significant increase in hydrogen peroxide levels, rising by 1.2 to 1.4 times in salvia and 1.5 to 1.8 times in oregano (Figure 1). In oregano, selenium treatment reduced hydrogen peroxide levels under drought conditions. However, in salvia, selenium treatment resulted in a significant decrease in hydrogen peroxide levels only in control plants, whereas under severe drought stress, hydrogen peroxide content increased significantly. In hyssop, no significant changes in hydrogen peroxide levels were observed in response to either increased drought stress or selenium treatment.

**Figure 2:**
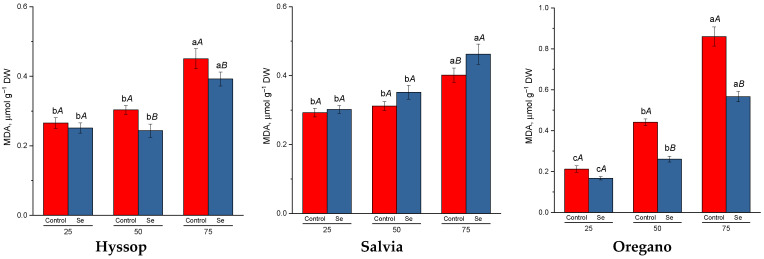
Effect of selenium on malondialdehyde (MDA) content under drought stress. Different lower-case letters indicate significant differences between plants by drought level for control, Se-treatment, and each species separately, and upper-case letters indicate significant differences between control and Se-treatment at *p* ≤ 0.05 based on post hoc Tukey’s test (*n* = 4).

Cultivating hyssop, salvia, and oregano under drought conditions resulted in the accumulation of malondialdehyde, a marker of oxidative stress, in the plants (Figure 2). In hyssop and salvia, significantly higher levels were detected only under severe drought conditions (75%), while in oregano, the increase in malondialdehyde was observed earlier, at moderate drought levels (50%). Selenium treatment under drought conditions led to a reduction in malondialdehyde content in hyssop and oregano by 1.1 to 1.2 times and 1.5 to 1.7 times, respectively. However, in salvia, selenium treatment caused an increase in malondialdehyde levels compared to untreated plants under the same conditions. 

### 2.4. Proline and Non-Enzymatic Antioxidants 

**Figure 3:**
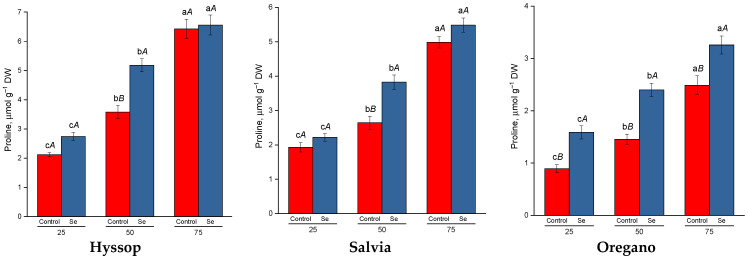
Effect of selenium on proline content under drought stress. Different lower-case letters indicate significant differences between plants by drought level for control, Se-treatment, and each species separately, and upper-case letters indicate significant differences between control and Se-treatment at *p* ≤ 0.05 based on post hoc Tukey’s test (*n* = 4).

A significant increase in proline content, by approximately 2.5 to 3.0 times, was found in all three studied species in response to the augmenting drought level (Figure 3). Treatment of plants with selenium provided an even greater increase in proline content compared to untreated plants. However, it should be noted that in hyssop and salvia, a significant increase was revealed only under moderate drought conditions. Treatment of oregano with selenium led to an increase in proline content in all examined variants including control plants.

**Figure 4:**
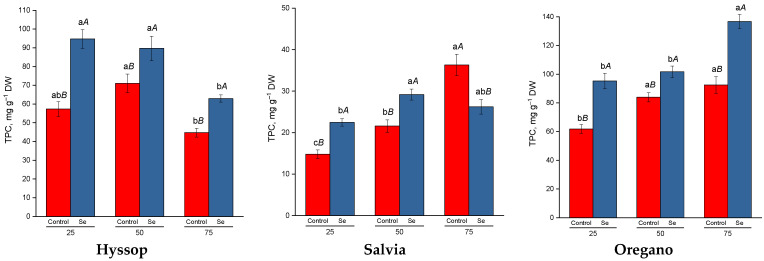
Effect of selenium on total phenolics content (TPC) under drought stress. Different lower-case letters indicate significant differences between plants by drought level for control, Se-treatment, and each species separately, and upper-case letters indicate significant differences between control and Se-treatment at *p* ≤ 0.05 based on post hoc Tukey’s test (*n* = 4).

The changes in the total content of phenolic compounds in response to increasing drought levels varied depending on the plant species (Figure 4). In hyssop, the highest phenolic compound content was observed under moderate drought conditions, while the lowest was detected at the highest drought level. In salvia, there was a linear increase in phenolic compound content as drought stress intensified. In oregano, phenolic compound content at moderate and severe drought levels was significantly higher than in the control, but there was no difference between these two stress levels. Selenium treatment stimulated the accumulation of phenolic compounds in both control plants and those subjected to drought. The exception was salvia under severe drought conditions, where selenium treatment led to a 1.4-fold decrease in phenolic compound content compared to untreated plants.

To evaluate the effects of drought and selenium on glutathione metabolism in the studied plants, we measured the content of both reduced and oxidized glutathione, as well as their ratio and total content (Table 4). Drought conditions in all three plant species resulted in a significant decrease in reduced glutathione and an increase in its oxidized form. However, the total glutathione content remained unchanged, while the ratio of reduced to oxidized glutathione significantly decreased. Selenium treatment did not affect glutathione redox metabolism under drought conditions, except in hyssop plants exposed to severe drought, where a significant decrease in oxidized glutathione content was observed.

The investigation of a drought’s impact on ascorbic acid metabolism revealed species-specific responses (Table 5). In hyssop and salvia, the highest content of reduced ascorbic acid was observed at moderate drought stress levels. In contrast, oregano showed a linear increase in ascorbic acid content as drought stress intensified. Additionally, the content of the oxidized form of ascorbic acid (dehydroascorbic acid) increased by 1.2 to 2.1 times in plants grown under drought conditions.

Selenium treatment led to an increase in ascorbic acid content, as well as the total content of ascorbic and dehydroascorbic acids, in all three species cultivated in control conditions (Table 5). However, the response of the plants to selenium treatment under drought conditions varied. In hyssop, no significant effect of selenium was observed. In salvia, selenium treatment under severe drought resulted in a decrease in ascorbic acid content and an increase in dehydroascorbic acid content compared to untreated plants. In oregano, selenium treatment significantly increased ascorbic acid content at moderate drought levels, while both dehydroascorbic acid and the total content of both forms increased significantly at moderate and severe drought levels.

### 2.5. Antioxidative Enzymes 

Drought cultivation led to increased superoxide dismutase (SOD) activity in hyssop and salvia by 2.5 and 1.5 times, respectively, whereas SOD activity in oregano remained unchanged (Table 6). Selenium treatment reduced SOD activity in hyssop under moderate drought and in salvia under severe drought. In contrast, selenium treatment caused a significant increase in SOD activity in oregano across all watering conditions.

Changes in catalase (CAT) activity under drought conditions varied by plant species (Table 6). Drought led to a decrease in CAT activity in hyssop and salvia by 1.6 to 2.1 times and 1.5 to 1.6 times, respectively, while in oregano, enzyme activity increased by 1.6 to 2.6 times. Selenium treatment significantly increased CAT activity in hyssop under moderate drought and in oregano under both moderate and severe drought conditions. However, selenium treatment did not have a significant effect on CAT activity in salvia.

The activity of ascorbate peroxidase (APX) in hyssop remained unchanged regardless of the drought level (Table 6). In contrast, salvia and oregano exhibited the highest APX activity under severe drought conditions, with APX activity in oregano being 1.5 times higher than in control plants. Selenium treatment led to a decrease in APX activity in salvia under control conditions but an increase in activity in both salvia and oregano under drought stress.

Changes in glutathione peroxidase (GPX) activity in response to drought were species-specific (Table 6). In hyssop, GPX activity increased sharply by 2.7 to 5.1 times, while in salvia, it decreased dramatically by 1.7 to 4.7 times under the same conditions. In oregano, a reduction in GPX activity was observed only under severe drought, with a decrease of 1.4 times. Selenium treatment stimulated GPX activity in all studied species, both in control plants and those grown under drought conditions.

Peroxidase (POD) activity increased in hyssop and oregano under drought conditions, while in salvia, POD activity remained unaffected by watering conditions (Table 6). 

Selenium treatment led to an increase in POD activity in hyssop in control conditions and in salvia grown under severe drought conditions. However, in oregano in control and moderate drought stress conditions, as well as in hyssop under severe drought stress, selenium treatment caused a decrease in POD activity.

### 2.6. Cluster Analysis

**Figure 5:**
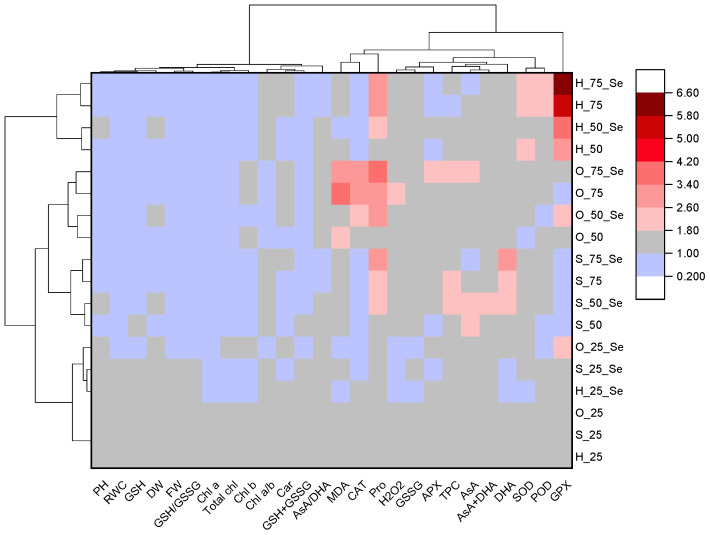
Heat map with clusters for the studied parameters (at the top) and *Lamiaceae* species cultivated under different levels of drought without and with Se-treatment (on the left). PH, plant height; FW, fresh weight; DW, dry weight; RWC, relative water content; MDA, malondialdehyde; H_2_O_2_, hydrogen peroxide; Chl *a*, chlorophyll *a*; Chl *b*, chlorophyll *b*; Chl *a*/*b*, chlorophyll *a*/*b* ratio; Total chl, total chlorophylls; Car, carotenoid; Pro, proline; GSH, reduced glutathione; GSSG, oxidized glutathione; AsA, ascorbic acid; DHA, dehydroascorbic acid; TPC, total phenolic compounds; SOD, superoxide dismutase; CAT, catalase; APX, ascorbate peroxidase; GPX, glutathione peroxidase; POD, peroxidase. H_75, H_75_Se, H_50, H_50_Se, H_25, H_25_Se, S_75, S_75_Se, S_50, S_50_Se, S_25, S_25_Se and O_75, O_75_Se, O_50, O_50_Se, O_25, O_25_Se, hyssop, salvia, and oregano plants cultivated at 75%, 50%, and 25% of drought stress without and with Se-treatment, respectively.

To construct the heat map and perform cluster analysis, the results were normalized to the control value (25% drought, no selenium treatment) separately for each plant species. In addition to the control group, cluster analysis identified four additional plant groups (Figure 5). 

The first group consisted of selenium-treated hyssop, salvia, and oregano plants grown under non-stress conditions. For this group, most of the measured indicators were similar to those of the control plants.

The second group comprised salvia plants grown under drought conditions, both with and without selenium treatment. This group was characterized by a significant increase in dehydroascorbic acid, ascorbic acid, and their total content.

The third group consisted of oregano plants, which were characterized by elevated malondialdehyde content, an increased AsA/DHA ratio, and higher CAT activity.

The fourth group, the most distinct from all the others, comprised of hyssop plants cultivated under drought conditions, including both selenium-treated and untreated plants. This group was notable for a sharp increase in the activities of GPX, SOD, and POD enzymes compared to the control plants.

## 3. Discussion

### 3.1. Differences in the Response of Hyssop, Salvia, and Oregano to Drought Stress

Drought, as one of the most significant abiotic stresses, has a detrimental impact on plants. Natural populations of aromatic plants from the Lamiaceae family are found in regions such as the Mediterranean, the Massif Central in France, the Alps, the Pyrenees, the Carpathians, the Balkan Peninsula, and the Crimean Peninsula [28]. These regions are characterized by water scarcity, high solar insolation, and elevated temperatures, particularly in summer—conditions that contribute to drought stress [29]. Such environmental factors can negatively affect the growth, yield, and quality of aromatic Lamiaceae plants. However, many of these plants possess adaptive mechanisms that enable them to respond to drought stress through morphological, anatomical, physiological, biochemical, and molecular changes, which help them mitigate the effects of stress and enhance their resistance [4]. At the same time, the response to drought stress and the level of resistance can be highly species-specific, varying significantly even within the same genus [30,31,32]. 

This study focused on three species—hyssop, salvia, and oregano—that differ in their drought resistance based on the literature data. Drought stress had the least impact on hyssop’s growth, with a 35% reduction in fresh weight and a 15% decrease in dry weight under severe drought conditions. As a typical xerophyte, hyssop exhibits high resistance to water scarcity [5]. In contrast, salvia and oregano, which are more sensitive to drought, showed a larger decrease in fresh weight (45–50%) and dry weight (30–40%) under similar conditions.

The reduction in plant productivity under drought stress can be attributed to several factors, many of which are related to changes in the functioning of the photosynthetic apparatus. For example, drought may reduce the absorption of photosynthetically active radiation due to delayed leaf expansion, temporary wilting or twisting of leaves during periods of high stress, and premature leaf senescence. Additionally, drought stress can impair the efficiency of utilizing absorbed photosynthetically active radiation for the production of new dry matter [33]. In our study, drought conditions in hyssop and salvia resulted in a reduction in chlorophylls *a* and *b*, as well as their total content in the leaves. This decline in chlorophyll content under water-deficient conditions may be linked to increased chlorophyllase activity, as its gene expression is known to be induced by stress. Furthermore, the inhibition of chlorophyll biosynthesis enzymes during the light stage and the degradation of chlorophylls by ROS, which are generated under stress, could also contribute to this decrease [34,35,36]. It is important to note that one of the plant adaptation mechanisms to drought involves regulating the size of the light-harvesting complex, which impacts the chlorophyll *a*/*b* ratio [37]. In our study, only oregano showed a reduction in this ratio by 1.3 to 1.5 times compared to control plants under drought conditions.

During drought stress, the production of ROS typically intensifies in various cellular compartments of plants [38]. In this study, a significant increase in hydrogen peroxide content was observed in salvia and oregano under drought conditions. However, in hyssop, no heightened hydrogen peroxide production was detected compared to control plants under water deficit. Despite this, all three species showed increased levels of malondialdehyde, a marker of lipid peroxidation, under drought stress. In addition to their pro-oxidant role, ROS are also involved in signal transduction, initiating a wide range of molecular, biochemical, physiological, and morphological responses to stress [14]. A cDNA microarray study identified in *Arabidopsis* 113 genes that were upregulated and 62 that were downregulated by H_2_O_2_, highlighting its key role in modulating plant drought responses. This includes the regulation of Ca^2+^ signaling, MAPK cascades, and gene expression [39].

Plants under water stress have developed several mechanisms to mitigate this challenge. The antioxidant system and osmotic regulation are the primary defense mechanisms that enhance crop resistance under such conditions [40]. Proline, a key osmolyte and signaling molecule, predominantly accumulates in the cytosol and plays a crucial role in protecting membranes and proteins, including enzymes, against various stresses [41]. In this study, a linear increase in proline content was observed as drought stress levels intensified across all three species. These findings are consistent with previous research showing that proline accumulation in response to drought stress is a common adaptive response in a variety of species, including members of the Lamiaceae family [42].

In addition to osmoprotectants, the antioxidant system plays a crucial role in protecting plants from drought stress. This system includes non-enzymatic antioxidants such as ascorbic acid, glutathione, phenolic compounds, and tocopherols, as well as antioxidant enzymes like SOD, CAT, APX, and GPX [14,43]. Our experiments demonstrated that the effects of drought stress on this system were species-specific. In salvia and oregano, there was an increase in the total content of phenolic compounds, ascorbic acid, and dehydroascorbic acid in response to increasing drought stress levels. In contrast, hyssop showed maximum values for these indicators only at moderate drought stress. Under severe drought, hyssop exhibited a sharp increase in GPX activity (by 5-fold), along with an increase in SOD and POD activities (by 2.5-fold). Meanwhile, in salvia, the activity of antioxidant enzymes either decreased significantly (GPX by 4.7-fold and CAT by 1.5-fold) or remained similar to control levels (POD, APX, SOD), depending on the drought intensity. In oregano, the most notable change was a 2.6-fold increase in CAT activity compared to the control under drought stress. Previous studies have shown that the activation of specific antioxidant components under drought stress depends on the stress level, duration, and plant species. Differences have been observed between drought-sensitive and drought-tolerant species, with some drought-sensitive plants exhibiting only moderate induction or even down-regulation of certain antioxidant system components [44].

Responses to drought stress are extremely different according to the genetic background. In fact, inter- and intra-species variations in drought resistance are known [45]. RWC is one of the most reliable and widely used indicators for defining both the sensitivity and the tolerance to water stress in plants [46]. In our experiments, drought stress reduced RWC in all three species, namely in hyssop by 7.5 to 17.5%, in salvia by 7.8 to 11.6%, and in oregano by 14.4 to 28.8%. Based on these results, it can be concluded that oregano is a more drought-sensitive species compared to hyssop and salvia. However, it is worth noting that RWC may not be considered a reliable indicator of drought resistance for all species. Previous experiments on rye and wheat have shown that RWC does not correlate with the stress tolerance index [47,48]. This could explain the lack of differences in the changes in RWC between the more resistant hyssop and the more sensitive salvia under drought stress conditions. Another important indicator used as a measure of sensitivity and tolerance to water stress is proline content [49]. In our study, hyssop, being the more resistant species, exhibited a higher content of proline under control conditions (Supplements, Table S2F). Furthermore, under stress conditions, proline content in hyssop increased by 1.7 to 3.0 times, while in salvia and oregano, proline content increased by 1.4 to 2.6 and 1.6 to 2.8 times, respectively. It is also important to note the high glutathione content in hyssop under control conditions, which sharply decreased under drought conditions and was accompanied by a significant increase in glutathione peroxidase activity (Supplementary Materials Table S2H). GSH plays an important role in the antioxidant and glyoxalase systems that are vital for ROS and methylglyoxal detoxification, respectively [50]. Previously, it was shown that methylglyoxal (MG) homeostasis played an essential role in promoting plant growth, development, metabolic adaptation, signal transduction, and thereby responses to drought stress [51]. Thus, the higher drought stress resistance of hyssop may be related to the more intense biosynthesis of glutathione and its more active involvement in the cell’s protective systems under stress conditions. Recent experimental data suggest that drawing conclusions about the sensitivity or resistance of plants to drought stress based on a single indicator (e.g., RWC) is not accurate. Generally, it is necessary to consider agronomic traits, as well as physiological, biochemical, and molecular markers for a comprehensive assessment. In our study, the cluster analysis conducted based on 25 indicators revealed that the response of hyssop to drought stress differed from that of salvia and oregano. However, for a more accurate interpretation, additional studies aimed at identifying differences among the studied species using molecular technologies are needed. Furthermore, expanding the research subjects to include various genotypes of a single species will allow for a deeper understanding of the mechanisms of drought stress resistance in the Lamiaceae family.

### 3.2. Features of the Selenium Effect on Growth and Biochemical Changes in Hyssop, Salvia, and Oregano Under Drought Stress Conditions

It is well established that most plants, including medicinal and aromatic species of the Lamiaceae family, are non-accumulators of selenium [52]. In this study, foliar treatment of hyssop, salvia, and oregano with selenium resulted in a significant increase in selenium content in all three species. Interestingly, the drought stress level did not influence the plants’ ability to accumulate selenium, contrasting with previous findings in sunflowers, where increased drought levels led to more active selenium absorption [53].

Although selenium treatment did not have a noticeable effect on growth parameters—such as height, fresh, and dry weight—in hyssop, salvia, and oregano grown under drought stress, biochemical changes were observed. Specifically, under moderate drought stress, the RWC in hyssop and salvia increased in response to selenium treatment. Similar results have been reported in other species, such as *Triticum aestivum*, *Trifolium repens*, *Sorghum*, and *Fragaria* × *ananassa* Duch. [24,54].

Selenium treatment under drought conditions had a significant impact on the levels of hydrogen peroxide, malondialdehyde, and chlorophylls in the plants. In hyssop and oregano, foliar selenium treatment during drought led to reduced levels of hydrogen peroxide and malondialdehyde, alongside an increase in chlorophyll *a* content compared to untreated plants. However, in salvia, particularly under severe drought stress, selenium treatment resulted in elevated levels of hydrogen peroxide and malondialdehyde but a decrease in chlorophyll content. Several studies have shown that selenium can enhance plant defense systems by detoxifying intracellular free radicals and boosting both enzymatic and non-enzymatic antioxidant activities, helping plants mitigate ROS and oxidative stress [55]. The observed differences in salvia’s response, though inconsistent with previous findings, could be attributed to species-specific reactions to selenium, particularly in non-accumulator plants, where selenium may act as a pro-oxidant [56]. In salvia, it is possible that selenium treatment exacerbated oxidative stress under severe drought conditions. However, further research on selenium metabolism in salvia is necessary to better interpret these results.

All species studied showed an increase in proline and phenolic compound accumulation in response to selenium treatment. Previous studies have demonstrated that selenium treatment can stimulate proline biosynthesis under stress conditions by increasing glutamyl kinase activity and reducing proline oxidase activity [56]. Additionally, selenium treatment in water-deficient plants has been found to enhance the activity of phenylalanine-ammonia-lyase, a key enzyme involved in the biosynthesis of phenolic compounds [57]. 

This study found that selenium treatment did not significantly alter glutathione metabolism in any of the three plant species. However, selenium did influence the levels of ascorbic and dehydroascorbic acids, particularly in salvia and oregano. Additionally, an increase in APX activity was observed in these species under drought stress conditions. Previous research has shown that APX activity can either increase or remain unchanged in response to selenium treatment, depending on the plant species, genotype, selenium concentration, and the form applied. It is worth noting that the regulation of APX activity may occur at both the transcriptional level, through increased gene expression, and at the post-translational level, via the replacement of Cys with SeCys during APX synthesis [58]. Despite the absence of a significant effect of selenium on glutathione redox metabolism in all three species, GPX activity was higher in selenium-treated plants compared to untreated ones. This may be due to selenium activating various signaling pathways that affect the expression of not only the genes encoding GPX but also glutathione reductase (GR), which aids in GSH regeneration. Furthermore, selenium treatment has been shown to increase the activity of both GPX and GR in plants under water deficiency conditions [59]. It is worth noting that for some plant species, an increase in the activity of enzymes involved in the regeneration of oxidized forms of glutathione and ascorbate has been shown (in particular, MDHAR, DHAR, and GR) [60], while changes in the activity of APX, GPX and in the level of substrates (AsA and GSH) may not be observed. Some studies demonstrate that certain conditions in different plant species activate different detoxification mechanisms within the AsA-GSH cycle, possibly due to the chemical form and concentration of applied Se and the plant species itself [58,61,62]. In addition, selenium metabolism and its transformation into organic forms in plants occur with the participation of glutathione [63]. Therefore, the peculiarities of the effect of selenium on the AsA-GSH cycle in different plant species may be associated with different intensities and rates of transformations of inorganic forms of selenium into organic ones. In this regard, for a more correct interpretation of the data obtained, it is necessary to conduct additional studies, both using different forms and concentrations of selenium, and studying the genes and enzymes involved in selenium metabolism.

## 4. Materials and Methods

### 4.1. Experimental Design and Plant Material

The study was conducted as a pot experiment under greenhouse conditions in the Institute of Living Systems at Immanuel Kant Baltic Federal University (Kaliningrad, Russia) in 2023–2024. For the experiment, seeds of three Lamiaceae plants (*Hyssopus officinalis* L. cv Lekar; *Salvia officinalis* L. cv. Kubanec; *Oregano vulgare* L. cv. Feya) were used. The seeds were sown into seed trays in the greenhouse. After germination, seedlings were grown for two months. Homogeneous seedlings of each species were transplanted into culture pots filled with the soil having the following physicochemical properties: soil texture—clay clam, pH—6.8, organic matter—2.21%, total N content—0.27%, available P—279 mg kg^−1^, available K—206 mg kg^−1^. The pots were 29 cm in height and 20 cm in radius. Each pot contained 10 kg of soil. There were four plants in each pot. During the growing experiment, the following conditions were maintained: humidity 70%; day—22 °C, 16 h; night—18 °C, 8 h; flux density of photosynthetically active radiation quanta—320 μmol/(m^2^·s).

After transplantation to pots, plants were grown for two weeks under normal irrigation (75% of the soil field capacity (SFC)) for root adaptation and stabilization in the soil. Following this acclimatization period, the experiment involving drought stress and selenium-treatment began. 

Drought stress was applied using a reduced irrigation strategy. Treatments were subjected to three levels of irrigation: (i) control—plants were irrigated up to 75% of SFC (this corresponds to 25% of the drought stress level); (ii) moderate drought stress—plants were irrigated up to 50% of SFC (this corresponds to 50% of the drought stress level); (iii) severe drought stress—plants were irrigated up to 25% of SFC (this corresponds to 75% of the drought stress level). The weight method was used to calculate the SFC. The pots were weighed every day, and the plants were irrigated with distilled water up to the corresponding target SFC.

The foliar treatment of plants with selenium was carried out using a hand atomizer once a week. As a source of selenium, an aqueous solution of sodium selenate (Na_2_SeO_4_) was used. The concentration of selenium was 50 μM. This concentration of selenium was selected based on a preliminary study on the effect of selenium on growth and secondary metabolite accumulation in hyssop, salvia, and oregano.

The factors of the experiment consisted of: (i) three plant species (hyssop, salvia, oregano); (ii) three levels of drought stress (25% (control), 50% (moderate drought stress), and 75% (severe drought stress)); (iii) foliar application of selenium (control and 50 μM). The experiment was conducted in a completely randomized design with four replications.

The plants were exposed to drought stress and selenium treatment for four weeks and then the harvesting was performed.

### 4.2. Determination of Growth Characteristics and Relative Water Content

On the day of plant collection, shoot height and fresh weight were measured. To determine dry weight, shoots were dried at 75 °C for 48 h and then weighed.

To determine the relative water content (RWC%), leaves were cut at the base of the blade at the time of harvesting the experimental plants. The weight of the fresh leaves was determined. Then, they were soaked in distilled water for 24 h at low light and low temperature (4 °C) levels. Following this, the leaves were removed from the water, blotted with a filter cloth, and the turgid weight was determined. The dry weight was measured after drying at 75 °C for 48 h. RWC% was calculated according to the formula presented in [64].

### 4.3. Determination of Selenium Concentration in Plant

Selenium concentration in plants was determined by hydride generation atomic absorption spectrometry (SpectrAA 220 FS, Varian, Palo Alto, CA, USA). The plant material, dried at 40 °C and crushed, was mineralized using a mixture of HNO_3_ and HClO_4_ acids. Se (VI) was reduced to Se (IV) with 6M HCl. Hydride generation was performed using NaBH_4_. The flow rate of fine gas (argon) was 200 mL per minute, and the temperature of the quartz cuvette was 900 °C. The intensity of light absorption was measured at a wavelength of 196.0 nm [65].

### 4.4. Determination of the Content of Photosynthetic Pigments

The content of photosynthetic pigments (chlorophyll *a*, chlorophyll *b*, and carotenoids) was determined in plant samples frozen in liquid nitrogen and stored at −80 °C by spectrophotometry after extraction with 80% acetone. Optical absorption of the extracts was measured at wavelengths of 663 nm, 645 nm, and 470 nm (UV-3600, Shimadzu, Tokyo, Japan). The concentration of photosynthetic pigments was calculated using the formulas presented in [66].

### 4.5. Determination of Oxidative Stress Parameters

Hydrogen peroxide (H_2_O_2_) content was determined in plant samples frozen in liquid nitrogen and stored at −80 °C by spectrophotometry using potassium iodide (KI) according to the protocol described previously [67]. Hydrogen peroxide concentration was calculated from the standard curve and converted to gram dry weight.

The determination of malondialdehyde (MDA) content was carried out by measuring the products of its reaction with thiobarbituric acid (TBA) using the spectrophotometry method. A weighed portion of plant samples frozen in liquid nitrogen and stored at −80 °C (0.3 g) was homogenized with 0.1% trichloroacetic acid (TCA). The homogenate was centrifuged at 1000 g for 10 min, and then 0.5% TBA in 20% TCA was added to the supernatant. The mixture was heated in a boiling water bath for 30 min, then it was very quickly cooled in ice and centrifuged at 1000 g for 15 min. The optical density in the samples was measured at wavelengths of 532 and 600 nm. The MDA concentration was calculated after subtracting the non-specific absorbance at 600 nm using the extinction coefficient ε = 1.56 × 10^5^ M^−1^ cm^−1^ [68].

### 4.6. Determination of Proline and Non-Enzymatic Antioxidant Content

Free proline was extracted twice from a weighed portion (0.3 g) of plant material frozen in liquid nitrogen and stored at −80 °C with 3% sulfosalicylic acid. The homogenate was centrifuged at 1000× *g* for 15 min. The proline content in the supernatant was determined with the ninhydrin reagent as described previously [56]. 

The total content of phenolic compounds was determined in lyophilized and crushed plant materials by spectrophotometry using the Folin–Ciocalteu reagent. The extraction of phenolic compounds was performed with a 70% aqueous solution of ethanol three times. Optical absorption was measured at a wavelength of 765 nm. Gallic acid was used as a standard [69]. The total content of phenolic compounds was expressed as mg of gallic acid equivalents per gram of dry weight.

The content of reduced and oxidized glutathione was determined in plant samples frozen in liquid nitrogen and stored at −80 °C by spectrophotometry according to the protocol presented in [70]. The content of oxidized glutathione was determined using the oxidation reaction of 5,5′-dithio-bis (2-nitrobenzoic acid). Then, the total content of glutathione was determined by the enzymatic reduction in oxidized glutathione with NADPH in the presence of glutathione reductase. The content of reduced glutathione was calculated as the difference between the total content of glutathione and oxidized glutathione. The content of glutathione was expressed in μmol per gram of dry weight.

The determination of ascorbic acid and its oxidized form (dehydroascorbic acid) was carried out in plant samples frozen in liquid nitrogen and stored at −80 °C by spectrophotometry according to the study [71]. The homogenization of plant material was carried out in 5% metaphosphoric acid. Determination of ascorbic acid was based on its ability to reduce Fe^3+^ to Fe^2+^, which in a complex with 2,2′-bipyridyl gives a colored compound with an absorption maximum at 530 nm wavelength. Further, dehydroascorbic acid was determined by its ability to interact with 2,4-dinitrophenylhydrazine in the presence of 85% sulfuric acid with the production of a colored compound with an absorption maximum at 540 nm wavelength.

### 4.7. Determination of Antioxidative Enzyme Activity

To determine the activity of antioxidant enzymes, the plant samples frozen in liquid nitrogen and stored at −80 °C were homogenized in an ice-cooled mortar using 10 mM potassium phosphate buffer (pH 7.0) containing 1% polyvinylpyrrolidone and 0.1 mM ethylenediaminetetraacetic acid (EDTA). To prevent enzyme inactivation during the determination of ascorbate peroxidase activity, 1 mM ascorbic acid and 2 mM β-mercaptoethanol were added to the homogenization buffer [60]. The homogenate was centrifuged at 15,000× *g* for 15 min at 4 °C. The supernatant was used for enzyme activity measuring.

SOD activity was determined by its ability to inhibit the photochemical reduction in nitroblue tetrazolium according to a previously described protocol [72]. CAT activity was determined by the decrease in optical absorption at a wavelength of 240 nm due to the decomposition of hydrogen peroxide and was calculated using an extinction coefficient of 39.4 mM^−1^ cm^−1^ [73]. APX activity was measured by the decrease in absorbance at a wavelength of 290 nm related to the oxidation of ascorbate with hydrogen peroxide and calculated using an extinction coefficient of 2.8 mM^−1^ cm^−1^ [74]. GPX activity was assessed by the decrease in reduced glutathione due to its oxidation by hydrogen peroxide [75]. POD activity was determined by spectrophotometry using guaiacol and hydrogen peroxide as substrates and calculated using an extinction coefficient of 26.6 mM^−1^ cm^−1^ (at 470 nm) [76].

To convert the activity of all enzymes per mg of protein, the protein concentration in samples was measured by spectrophotometry using the Bradford assay [77]. Bovine serum albumin was used as a standard.

### 4.8. Statistical Analysis

All the biochemical assays were carried out in triplicate. Statistical analysis of data were performed only with biological replications corresponding to the number of pots in each treatment (*n* = 4). The obtained experimental data were analyzed using a three-way ANOVA, where factor 1 was the plant species; factor 2 was the drought level; factor 3 was selenium treatment (Supplementary Materials Table S1). Since a significant relationship between a species and drought level was revealed on the studied biochemical parameters, the experimental data were additionally processed using a two-way ANOVA, where factor 1 was the drought level; factor 2 was selenium treatment, separately for each species. The significance of differences between means was determined by the post hoc Tukey’s test at *p* ≤ 0.05. An analysis of variance was performed using the software SigmaPlot 12.5 (Systat Software GmbH, Erkrath, Germany). Plotting of graphs, heat maps, and cluster analysis were performed using OriginPro 2019b (OriginLab Corporation, Northampton, MA, USA). The Euclidean distance was used as a measure of similarity in cluster analysis.

## 5. Conclusions

The study highlighted differences in the drought stress response among three plant species from the Lamiaceae family: hyssop, salvia, and oregano. Under drought conditions, hyssop exhibited a significant increase in GPX, SOD, and POD activities, oregano showed enhanced CAT activity, and salvia experienced a sharp increase in ascorbic acid content under moderate stress. Selenium treatment of all three species in drought-free conditions did not lead to notable changes in growth or biochemical characteristics. However, under drought stress, foliar selenium treatment stimulated the accumulation of proline and phenolic compounds and increased GPX activity in hyssop, salvia, and oregano. Based on the outcome of the present investigation, the foliar application of selenium to the hyssop, salvia, and oregano cultivated in arid and semiarid areas and regions suffering from temporary drought can be recommended. However, further studies aimed at evaluating the metabolism of selenium compounds in each of the studied species and exploring how selenium regulates biochemical processes at the transcriptomic and post-translational levels are required. The latter will provide new insights into the molecular mechanisms responsible for differences in the response of closely related plant species to drought and selenium treatment.

## Figures and Tables

**Table 1:** Effect of Selenium on Plant Growth under Drought Stress.

Level of Drought	Plant Height [cm]		Fresh Weight [g]		Dry Weight [g]		RWC ^1^ [%]	
	Control	Se	Control	Se	Control	Se	Control	Se
Hyssop								
25%	23.46 ± 1.04 a	23.60 ± 1.47 a	5.32 ± 0.25 a	5.77 ± 0.29 a	0.58 ± 0.03 a	0.65 ± 0.03 a	80.06 ± 0.68 a	80.20 ± 0.57 a
50%	23.17 ± 1.09 a	24.34 ± 1.45 a	4.81 ± 0.27 a	5.01 ± 0.25 a	0.57 ± 0.02 a	0.63 ± 0.03 a	74.14 ± 0.61 b	78.68 ± 0.59 a *
75%	16.41 ± 1.24 b	18.07 ± 1.20 b	3.44 ± 0.21 b	2.92 ± 0.18 b	0.50 ± 0.02 a	0.41 ± 0.01 b	66.09 ± 0.33 c	64.87 ± 0.94 b
Salvia								
25%	40.10 ± 2.25 a ^2^	42.86 ± 2.87 a	8.47 ± 0.29 a	8.58 ± 0.27 a	0.93 ± 0.07 a	0.96 ± 0.03 a	77.43 ± 0.67 a	77.54 ± 0.39 a
50%	35.53 ± 2.61 ab	40.63 ± 2.80 ab	6.88 ± 0.36 b	7.51 ± 0.28 b	0.85 ± 0.06 a	0.94 ± 0.03 a	71.98 ± 0.35 b	76.54 ± 0.34 a *
75%	29.91 ± 1.58 b	31.91 ± 0.99 b	4.63 ± 0.30 c	4.72 ± 0.19 c	0.55 ± 0.03 b	0.63 ± 0.02 b	68.71 ± 0.81 c	69.38 ± 0.55 b
Oregano								
25%	52.78 ± 2.98 a	54.41 ± 2.46 a	9.41 ± 0.49 a	8.87 ± 0.48 a	1.65 ± 0.11 a	1.75 ± 0.14 a	90.13 ± 0.47 a	88.13 ± 0.64 a *
50%	42.24 ± 2.43 b	43.19 ± 3.09 b	8.25 ± 0.55 a	8.52 ± 0.60 a	1.55 ± 0.10 a	1.80 ± 0.12 a	77.05 ± 0.39 b	75.84 ± 1.06 b
75%	35.77 ± 1.72 b	36.75 ± 1.61 b	4.63 ± 0.31 b	5.10 ± 0.24 b	1.11 ± 0.07 b	1.03 ± 0.08 b	64.95 ± 0.74 c	63.07 ± 0.45 c

^1^ RWC, relative water content. ^2^ Different letters indicate significant differences between plants by drought level for control, Se-treatment, and each species separately, and asterisks * indicate significant differences between control and Se-treatment at *p* ≤ 0.05 based on post hoc Tukey’s test (*n* = 4).

**Table 2:** Selenium concentration in plants (μg g^−1^ DW).

Level of Drought	Hyssop		Salvia		Oregano	
	Control	Se	Control	Se	Control	Se
25%	0.024 ± 0.001 a ^1^	9.53 ± 0.61 a *	0.0504 ± 0.003 a	2.93 ± 0.17 a *	0.184 ± 0.011 a	3.36 ± 0.16 a *
50%	0.024 ± 0.001 a	8.89 ± 0.56 a *	0.0522 ± 0.002 a	2.94 ± 0.16 a *	0.197 ± 0.007 a	3.22 ± 0.21 a *
75%	0.023 ± 0.001 a	7.86 ± 0.24 a *	0.0489 ± 0.002 a	3.30 ± 0.12 a *	0.173 ± 0.011 a	2.71 ± 0.15 a *

^1^ Different letters indicate significant differences between plants by drought level for control, Se-treatment, and each species separately, and asterisks * indicate significant differences between control and Se-treatment at *p* ≤ 0.05 based on post hoc Tukey’s test (*n* = 4).

**Table 3:** Effect of Selenium on Photosynthetic Pigments under Drought Stress.

Level of Drought	Chl *a* [mg g^−1^ DW ^1^]		Chl *b* [mg g^−1^ DW]		Chl *a*/*b*		Total Chl [mg g^−1^ DW]		Car [mg g^−1^ DW]	
	Control	Se	Control	Se	Control	Se	Control	Se	Control	Se
Hyssop										
25%	13.13 ± 0.63 a ^2^	12.92 ± 0.44 a	6.17 ± 0.18 a	5.83 ± 0.11 a	2.14 ± 0.16 b	2.22 ± 0.11 a	19.30 ± 0.50 a	18.75 ± 0.37 a	2.76 ± 0.16 a	2.87 ± 0.09 a
50%	9.95 ± 0.29 b	12.25 ± 0.33 a *	3.66 ± 0.12 b	4.32 ± 0.12 b *	2.73 ± 0.13 a	2.84 ± 0.13 a	13.61 ± 0.29 b	16.58 ± 0.32 b *	2.67 ± 0.19 a	2.54 ± 0.20 a
75%	8.13 ± 0.26 c	9.38 ± 0.52 b	3.06 ± 0.13 c	3.66 ± 0.12 c *	2.67 ± 0.13 ab	2.58 ± 0.22 a	11.20 ± 0.29 c	13.04 ± 0.43 c *	3.13 ± 0.26 a	2.88 ± 0.12 a
Salvia										
25%	8.35 ± 0.42 a	8.30 ± 0.32 a	3.74 ± 0.13 a	3.62 ± 0.25 a	2.23 ± 0.13 a	2.33 ± 0.22 a	12.09 ± 0.45 a	11.93 ± 0.31 a	2.34 ± 0.02 a	2.16 ± 0.13 ab
50%	6.18 ± 0.18 b	7.55 ± 0.12 a *	2.58 ± 0.06 b	3.20 ± 0.05 a *	2.40 ± 0.10 a	2.36 ± 0.03 a	8.76 ± 0.16 b	10.74 ± 0.16 a *	2.14 ± 0.17 a	2.02 ± 0.13 b
75%	6.02 ± 0.36 b	4.89 ± 0.31 b *	2.34 ± 0.11 b	1.91 ± 0.13 b *	2.60 ± 0.26 a	2.56 ± 0.14 a	8.36 ± 0.28 b	6.79 ± 0.41 b *	2.31 ± 0.11 a	2.60 ± 0.15 a
Oregano										
25%	5.91 ± 0.31 a	5.87 ± 0.12 a	2.26 ± 0.14 a	2.31 ± 0.15 a	2.62 ± 0.02 a	2.57 ± 0.14 a	8.17 ± 0.45 a	8.18 ± 0.22 a	2.99 ± 0.15 ab	3.07 ± 0.15 a
50%	5.12 ± 0.26 ab	5.37 ± 0.18 ab	2.57 ± 0.13 a	2.15 ± 0.09 a	2.00 ±0.03 b	2.51 ± 0.10 a *	7.69 ± 0.39 a	7.52 ± 0.24 a	2.55 ± 0.10 b	3.23 ± 0.15 a
75%	4.46 ± 0.17 b	5.27 ± 0.16 b *	2.48 ± 0.06 a	2.40 ± 0.07 a	1.80 ±0.07 c	2.19 ± 0.09 b *	6.94 ± 0.19 a	7.66 ± 0.17 a	3.28 ± 0.17 a	3.22 ± 0.21 a

^1^ Chl *a*, chlorophyll *a*; Chl *b*, chlorophyll *b*; Total Chl, total chlorophylls; Chl *a*/*b*, chlorophyll *a*/*b* ratio; Car, carotenoids; DW, dry weight. ^2^ Different letters indicate significant differences between plants by drought level for control, Se-treatment, and each species separately, and asterisks * indicate significant differences between control and Se-treatment at *p* ≤ 0.05 based on post hoc Tukey’s test (*n* = 4).

**Table 4:** Effect of Selenium on Glutathione Content under Drought Stress.

Level of Drought	GSH [μmol g^−1^ DW ^1^]		GSSG [μmol g^−1^ DW]		GSH/GSSG		GSH + GSSG [μmol g^−1^ DW]	
	Control	Se	Control	Se	Control	Se	Control	Se
Hyssop								
25%	254.01 ± 14.94 a ^2^	273.21 ± 15.13 a	42.29 ± 0.86 b	41.25 ± 2.11 b	6.03 ± 0.47 a	6.64 ± 0.31 a	296.30 ± 14.22 a	314.46 ± 16.41 a
50%	233.77 ± 17.00 ab	245.86 ± 8.55 ab	50.10 ± 1.60 b	48.30 ± 1.79 b	4.66 ± 0.26 b	5.13 ± 0.36 b	283.88 ± 18.20 a	294.16 ± 6.88 a
75%	198.11 ± 5.64 b	208.87 ± 8.45 b	67.63 ± 4.05 a	60.07 ± 1.90 a *	2.97 ± 0.22 c	3.48 ± 0.14 c	265.73 ± 5.42 a	268.94 ± 9.41 a
Salvia								
25%	192.89 ± 13.57 a	207.96 ± 10.13 a	36.39 ± 2.05 b	38.18 ± 0.90 b	5.32 ± 0.33 a	5.45 ± 0.31 a	229.27 ± 14.89 a	246.13 ± 10.04 a
50%	194.67 ± 11.47 a	185.75 ± 8.57 a	44.02 ± 2.10 b	39.89 ± 1.84 b	4.44 ± 0.28 a	4.71 ± 0.40 a	238.70 ± 12.28 a	225.64 ± 7.39 ab
75%	160.65 ± 6.15 b	148.92 ± 3.09 b	59.77 ± 4.77 a	64.24 ± 1.64 a	2.74 ± 0.26 b	2.32 ± 0.03 b	220.42 ± 7.96 a	213.17 ± 4.57 b
Oregano								
25%	86.74 ± 3.92 a	76.05 ± 3.27 a	14.54 ± 0.96 b	13.66 ± 1.28 b	6.06 ± 0.53 a	5.66 ± 0.35 a	101.28 ± 4.01 a	89.72 ± 4.50 a
50%	77.42 ± 4.66 ab	78.54 ± 3.36 a	22.20 ± 1.16 a	20.89 ± 1.48 a	3.50 ± 0.21 b	3.83 ± 0.38 b	99.62 ± 5.26 a	99.42 ± 3.01 a
75%	67.41 ± 3.04 b	70.49 ± 3.57 a	24.87 ± 1.68 a	23.74 ± 1.61 a	2.77 ± 0.29 b	3.00 ± 0.22 b	92.29 ± 2.06 a	94.23 ± 4.48 a

^1^ GSH, reduced glutathione; GSSG, oxidized glutathione; DW, dry weight. ^2^ Different letters indicate significant differences between plants by drought level for control, Se-treatment, and each species separately, and asterisks * indicate significant differences between control and Se-treatment at *p* ≤ 0.05 based on post hoc Tukey’s test (*n* = 4).

**Table 5:** Effect of Selenium on Ascorbic Acid Content under Drought Stress.

Level of Drought	AsA [mg g^−1^ DW ^1^]		DHA [mg g^−1^ DW]		AsA/DHA		AsA + DHA [mg g^−1^ DW]	
	Control	Se	Control	Se	Control	Se	Control	Se
Hyssop								
25%	15.32 ± 0.81 b ^2^	21.28 ± 1.63 a *	6.72 ± 0.31 b	6.34 ± 0.41 b	2.30 ± 0.16 a	3.43 ± 0.45 a *	22.04 ± 0.85 c	27.62 ± 1.36 ab *
50%	25.17 ± 0.94 a	23.34 ± 1.24 a	11.22 ± 1.57 a	9.50 ± 0.58 a	2.41 ± 0.42 a	2.47 ± 0.15 ab	36.39 ± 0.96 a	32.84 ± 1.62 a
75%	16.47 ± 1.16 b	14.34 ± 1.51 b	11.34 ± 0.49 a	9.85 ± 0.65 a	1.46 ± 0.12 a	1.48 ± 0.18 b	27.82 ± 1.30 b	24.19 ± 1.59 b
Salvia								
25%	6.74 ± 0.35 b	9.59 ± 0.57 b *	3.54 ± 0.15 c	3.32 ± 0.21 c	1.92 ± 0.14 a	2.94 ± 0.34 a *	10.27 ± 0.36 b	12.93 ± 0.42 c *
50%	12.23 ± 0.65 a	12.45 ± 0.44 a	5.99 ± 0.36 b	6.47 ±0.32 b	2.05 ± 0.11 a	1.94 ± 0.12 b	18.22 ± 0.92 a	18.92 ±0.51 a
75%	8.31 ± 0.38 b	6.45 ± 0.42 c *	7.50 ± 0.32 a	9.84 ±0.28 a *	1.11 ± 0.07 b	0.64 ± 0.06 c	15.80 ± 0.50 a	16.30 ± 0.30 b
Oregano								
25%	18.77 ± 1.42 c	24.51 ± 1.20 b *	5.68 ± 0.24 b	6.16 ± 0.11 b	3.30 ± 0.16 b	3.98 ± 0.20 a	24.44 ± 1.63 c	30.67 ± 1.21 b *
50%	24.31 ± 1.51 b	29.64 ± 1.74 ab *	6.32 ± 0.23 ab	7.26 ± 0.45 ab *	3.84 ± 0.13 ab	4.16 ± 0.44 a	30.63 ± 1.72 b	36.90 ± 1.32 a *
75%	30.25 ± 1.19 a	33.95 ± 1.37 a	6.75 ± 0.26 a	7.99 ± 0.43 a *	4.52 ± 0.33 a	4.29 ± 0.29 a	37.00 ± 0.96 a	41.94 ± 1.40 a *

^1^ AsA, ascorbic acid; DHA, dehydroascorbic acid; DW, dry weight. ^2^ Different letters indicate significant differences between plants by drought level for control, Se-treatment, and each species separately, and asterisks * indicate significant differences between control and Se-treatment at *p* ≤ 0.05 based on post hoc Tukey’s test (*n* = 4).

**Table 6:** Effect of Selenium on the Activities of Antioxidant Enzymes under Drought Stress.

Level of Drought	SOD ^1^ [U mg^−1^ Protein]		CAT [µmol H_2_O_2_ mg^−1^ Protein min^−1^]		APX [µmol AsA mg^−1^ Protein min^−1^]		GPX [µmol GSH mg^−1^ Protein min^−1^]		POD [µmol Guaiacol mg^−1^ Protein min^−1^]	
	Control	Se	Control	Se	Control	Se	Control	Se	Control	Se
Hyssop										
25%	1.84 ± 0.05 b ^2^	1.69 ± 0.10 c	614.08 ± 50.91 a	636.06 ± 30.08 a	8.08 ± 0.45 a	8.41 ± 0.65 a	0.083 ± 0.006 c	0.144 ± 0.009 c *	0.83 ± 0.04 c	1.09 ± 0.06 b*
50%	3.97 ± 0.35 a	2.84 ± 0.15 b *	391.58 ± 28.61 b	507.24 ± 20.98 b *	7.19 ± 0.45 a	8.34 ± 0.59 a	0.224 ± 0.007 b	0.297 ± 0.020 b *	1.38 ± 0.05 b	1.23 ± 0.06 b
75%	4.42 ± 0.42 a	4.65 ± 0.31 a	291.46 ± 22.16 b	319.33 ± 11.33 c	6.78 ± 0.30 a	7.06 ± 0.41 a	0.426 ± 0.027 a	0.547 ± 0.015 a *	2.05 ± 0.11 a	1.66 ± 0.09 a *
Salvia										
25%	2.10 ± 0.09 b	2.18 ± 0.15 a	378.38 ± 25.37 a	359.64 ± 16.08 a	8.68 ± 0.43 ab	6.34 ± 0.37 b *	0.482 ± 0.038 a	0.695 ± 0.018 a *	2.70 ± 0.08 a	2.74 ± 0.16 b
50%	2.31 ± 0.11 b	2.31 ± 0.11 a	239.06 ± 14.63 b	266.09 ± 6.11 b	7.48 ± 0.46 b	10.09 ± 0.45 a *	0.277 ± 0.009 b	0.398 ± 0.028 b *	2.69 ± 0.11 a	2.86 ± 0.19 b
75%	3.04 ± 0.20 a	2.57 ± 0.14 a *	248.31 ± 10.80 b	219.63 ±16.31 b	9.65 ± 0.70 a	11.34 ± 0.48 a *	0.103 ±0.008 c	0.192 ± 0.013 c *	2.74 ± 0.16 a	4.00 ± 0.22 a *
Oregano										
25%	0.89 ± 0.05 a	1.13 ± 0.08 ab *	121.44 ± 6.25 c	114.02 ± 6.85 c	13.87 ± 0.71 b	15.58 ± 0.80 c	0.071 ± 0.005 a	0.131 ± 0.009 ab *	1.09 ± 0.07 b	0.73 ± 0.04 c *
50%	0.77 ± 0.03 a	1.00 ± 0.07 b *	191.36 ± 12.37 b	303.63 ± 23.13 b *	15.20 ± 1.14 b	22.24 ± 1.25 b *	0.084 ± 0.005 a	0.153 ± 0.007 a *	1.33 ± 0.05 ab	1.04 ± 0.07 b *
75%	0.94 ± 0.04 a	1.42 ± 0.08 a *	317.49 ± 20.78 a	386.62 ± 19.78 a *	21.49 ± 1.57 a	28.78 ± 1.71 a *	0.051 ± 0.004 b	0.122 ± 0.006 b *	1.53 ± 0.08 a	1.41 ± 0.08 a

^1^ SOD, superoxide dismutase; U, units; CAT, catalase; APX, ascorbate peroxidase; AsA, ascorbic acid; GPX, glutathione peroxidase; GSH, glutathione; POD, peroxidase. ^2^ Different letters indicate significant differences between plants by drought level for control, Se-treatment, and each species separately, and asterisks * indicate significant differences between control and Se-treatment at *p* ≤ 0.05 based on post hoc Tukey’s test (*n* = 4).

## Data Availability

The original contributions presented in this study are included in the article and Supplementary Materials. Further inquiries can be directed to the corresponding author.

## Supplementary Materials

The following supporting information can be downloaded at: https://www.mdpi.com/article/10.3390/plants13212986/s1, Table S1: Results of 3-factorial ANOVA; Table S2: Results of 2-factorial ANOVA.
