# Supplementary material for: Comparative Study on the Response of Hyssop (Hyssopus officinalis L.), Salvia (Salvia officinalis L.), and Oregano (Origanum vulgare L.) to Drought Stress Under Foliar Application of Selenium
Source: Plants (Basel). 2024 Oct 25;13(21):2986. doi: 10.3390/plants13212986 (PMC11547996; doi:10.3390/plants13212986)
Supplement: Supplementary file 1 [file plants-13-02986-s001.zip › Table S1.pdf]

Table S1-A: Results of 3-factorial ANOVA for growth characteristics and selenium concentration.

**Main effects/Factors**	Level of factors	**Plant height, cm**	**Fresh weight, g**	**Dry weight, g**	**RWC, %**	**Se concentration in plant, µg g^{-1}**
Species (S)	Hyssop	21.51 c	4.54 b	0.559 c	74.01 a	4.39 a
	Salvia	36.80 b	6.80 a	0.813 b	73.60 a	1.55 b
	Oregano	44.19 a	7.64 a	1.483 a	76.53 a	1.64 b
Drought (D)	25 % (control)	39.53 a	7.74 a	1.088 a	82.25 a	2.68 a
	50 %	34.82 ab	6.83 a	1.057 a	75.71 b	2.55 a
	75 %	28.14 b	4.24 b	0.711 b	66.18 c	2.35 a
Se treatment (Se)	Control	33.26 a	6.21 a	0.92 a	74.50 a	0.086 b
	50 µM	35.07 a	6.33 a	0.98 a	74.92 a	4.97 a
**Significance**	S	*	*	*	*	*
	D	*	*	*	*	ns
	Se	ns	ns	ns	ns	*
	S * D	*	*	*	*	*
	S * Se	ns	ns	ns	*	*
	D * Se	ns	ns	ns	*	ns
	S * D * Se	ns	ns	ns	*	*

Data was evaluated via three-way ANOVA, followed by Tukey HSD test (mean, n = 4). Identical letters indicate that values do not differ significantly. Asterisks indicate significantly influential factors. RWC, relative water content

Table S1-B: Results of 3-factorial ANOVA for oxidative stress parameters and photosynthetic pigments.

**Main effects**	Level of factors	**MDA, µmol g^{-1} DW**	**H_2O_2, µmol g^{-1} DW**	**Pro, µmol g^{-1} DW**	**Chl a, mg g^{-1} DW**	**Chl b, mg g^{-1} DW**	**Chl a/b**	**Total Chl, mg g^{-1} DW**	**Car, mg g^{-1} DW**
Species (S)	Hyssop	0.318 a	3.42 a	4.43 a	10.96 a	4.45 a	2.53 a	15.41 a	2.81 b
	Salvia	0.354 a	2.17 b	3.52 a	6.88 b	2.90 b	2.42 a	9.78 a	2.26 c
	Oregano	0.418 a	2.92 a	2.01 b	5.33 c	2.36 b	2.28 a	7.70 a	3.06 a
Drought (D)	25 % (control)	0.248 b	2.24 c	1.92 c	9.08 a	3.99 a	2.35 a	13.07 a	2.70 ab
	50 %	0.319 a	2.85 b	3.18 b	7.74 ab	3.08 ab	2.47 a	10.82 ab	2.53 b
	75 %	0.522 a	3.14 a	4.87 a	6.36 b	2.64 b	2.40 a	9.00 a	2.90 a
Se treatment (Se)	Control	0.393 a	2.99 a	2.95 a	7.47 a	3.21 a	2.36 a	10.68 a	2.69 a
	50 µM	0.333 a	2.68 a	3.70 a	7.98 a	3.27 a	2.46 a	11.24 a	2.73 a
**Significance**	S	*	*	*	*	*	*	*	*
	D	*	*	*	*	*	ns	*	*
	Se	*	*	*	*	ns	ns	*	ns
	S * D	*	*	*	*	*	*	*	ns
	S * Se	*	*	ns	*	*	ns	*	ns
	D * Se	*	ns	*	*	*	ns	*	ns
	S * D * Se	*	*	ns	*	*	ns	*	ns

Data was evaluated via three-way ANOVA, followed by Tukey HSD test (mean, n = 4). Identical letters indicate that values do not differ significantly. Asterisks indicate significantly influential factors. MDA, malondialdehyde; Pro, proline; Chl a, chlorophyll *a*; Chl b, chlorophyll *b;* Total Chl, total chlorophylls; Chl a/b, chlorophyll *a/b* ratio; Car, carotenoids

Table S1-C: Results of 3-factorial ANOVA for non-enzymatic antioxidants.

**Main effects**	Level of factors	**TPC, mg g^{-1} DW**	**GSH, µmol g^{-1} DW**	**GSSG, µmol g^{-1} DW**	**GSH/ GSSG**	**GSH + GSSG, µmol g^{-1} DW**	**AsA, mg g^{-1} DW**	**DHA, mg g^{-1} DW**	**AsA/ DHA**	**AsA + DHA, mg g^{-1} DW**
Species (S)	Hyssop	70.08 b	235.6 a	51.61 a	4.82 a	287.2 a	19.32 b	9.16 a	2.26 b	28.48 b
	Salvia	25.10 c	181.8 b	47.08 a	4.17 a	228.9 b	9.30 c	6.11 b	1.77 b	15.41 c
	Oregano	95.31 a	76.1 c	19.98 b	4.14 a	96.1 c	26.91 a	6.69 b	4.02 a	33.60 a
Drought (D)	25 % (control)	57.73 a	181.8 a	31.05 ab	5.86 a	212.9 a	16.04 a	5.29 b	2.98 a	21.33 b
	50 %	66.19 a	169.3 a	37.57 b	4.38 a	206.9 a	21.19 a	7.79 a	2.81 a	28.98 a
	75 %	66.58 a	142.4 a	50.05 a	2.88 a	192.5 a	18.30 a	8.88 a	2.25 a	27.18 a
Se treatment (Se)	Control	53.78 b	162.9 a	40.20 a	4.28 a	203.1 a	17.51 a	7.23 a	2.55 a	24.74 a
	50 µM	73.22 a	166.2 a	38.91 a	4.47 a	205.1 a	19.51 a	7.42 a	2.82 a	26.92 a
**Significance**	S	*	*	*	*	*	*	*	*	*
	D	*	*	*	*	*	*	*	*	*
	Se	*	ns	ns	ns	ns	*	ns	*	*
	S * D	*	*	*	*	ns	*	*	*	*
	S * Se	*	ns	ns	ns	ns	*	*	ns	*
	D * Se	*	ns	ns	ns	ns	*	ns	*	*
	S * D * Se	*	ns	ns	ns	ns	ns	ns	ns	*

Data was evaluated via three-way ANOVA, followed by Tukey HSD test (mean, n = 4). Identical letters indicate that values do not differ significantly. Asterisks indicate significantly influential factors. TPC, total phenolic compounds; DW, dry weight; GSH, reduced glutathione; GSSG, oxidized glutathione; AsA, ascorbic acid; DHA, dehydroascorbic acid

Table S1-D: Results of 3-factorial ANOVA for antioxidant enzymes.

**Main effects/Factors**	Level of factors	**SOD, U mg^{-1} protein**	**CAT, µmol H_2O_2 mg^{-1} Protein min^{-1}**	**APX, µmol AsA mg^{-1} protein min^{-1}**	**GPX, µmol GSH mg^{-1} protein min^{-1}**	**POD, µmol guaiacol mg^{-1} protein min^{-1}**
Species (S)	Hyssop	3.24 a	460.0 a	7.65 b	0.287 a	1.38 b
	Salvia	2.42 b	285.2 b	8.93 b	0.337 a	2.96 a
	Oregano	1.02 c	239.1 b	19.53 a	0.102 b	1.19 b
Drought (D)	25 % (control)	1.64 b	370.6 a	10.16 a	0.247 a	1.53 b
	50 %	2.20 ab	316.5 a	11.76 a	0.239 a	1.76 ab
	75 %	2.84 a	297.1 a	14.18 a	0.240 a	2.23 a
Se treatment (Se)	Control	2.25 a	310.4 a	10.94 a	0.187 b	1.82 a
	50 µM	2.20 a	345.8 a	13.13 a	0.298 a	1.86 a
**Significance**	S	*	*	*	*	*
	D	*	*	*	ns	*
	Se	*	*	*	*	ns
	S * D	ns	*	*	*	ns
	S * Se	*	*	*	*	*
	D * Se	ns	*	*	ns	*
	S * D * Se	*	ns	ns	*	*

Data was evaluated via three-way ANOVA, followed by Tukey HSD test (mean, n = 4). Identical letters indicate that values do not differ significantly. Asterisks indicate significantly influential factors. SOD, superoxide dismutase; U, units; CAT, catalase; APX, ascorbate peroxidase; AsA, ascorbic acid; GPX, glutathione peroxidase; GSH, glutathione; POD, peroxidase
