# Supplementary material for: Comparative Study on the Response of Hyssop (Hyssopus officinalis L.), Salvia (Salvia officinalis L.), and Oregano (Origanum vulgare L.) to Drought Stress Under Foliar Application of Selenium
Source: Plants (Basel). 2024 Oct 25;13(21):2986. doi: 10.3390/plants13212986 (PMC11547996; doi:10.3390/plants13212986)
Supplement: Supplementary file 1 [file plants-13-02986-s001.zip › Table S2.pdf]

Se-treatment: Control

Table S2-A: Results of 2-factorial ANOVA for growth characteristics and selenium concentration.

Level of drought	Species	PH	FW	DW	RWC	Se
25%	Hyssop	23.455 c	5.322 b	0.580 c	80.061 b	0.0236 c
	Salvia	40.097 b	8.476 a	0.930 b	77.433 c	0.0504 b
	Oregano	52.775 a	9.414 a	1.653 a	90.133 a	0.184 a
50%	Hyssop	23.168 b	4.811 c	0.571 c	74.141 b	0.0238 c
	Salvia	35.534 a	6.876 b	0.851 b	71.978 c	0.0522 b
	Oregano	42.235 a	8.253 a	1.547 a	77.047 a	0.197 a
75%	Hyssop	16.405 b	3.437 b	0.501 b	66.088 b	0.0228 c
	Salvia	29.911 a	4.626 a	0.555 b	68.706 a	0.0488 b
	Oregano	35.773 a	4.627 a	1.110 a	64.953 b	0.173 a
**Significance**	S	*	*	*	*	*
	D	*	*	*	*	ns
	S*D	ns	*	*	*	ns

Data was evaluated via two-way ANOVA, followed by Tukey HSD test (mean, n = 4). Identical letters indicate that values do not differ significantly. Asterisks indicate significantly influential factors. RWC, relative water content

Table S2-B: Results of 2-factorial ANOVA for oxidative stress parameters and photosynthetic pigments.

Level of drought	Species	MDA	H_2O_2	Pro	Chl a	Chl b	Chl a/b	Total Chl	Car
25%	Hyssop	0.265 a	3.254 a	2.119 a	13.126 a	6.172 a	2.139 b	19.297 a	2.763 ab
	Salvia	0.293 a	1.771 b	1.930 a	8.349 b	3.741 b	2.238 ab	12.090 b	2.340 b
	Oregano	0.212 b	2.226 b	0.894 b	5.911 c	2.260 c	2.620 a	8.171 c	2.991 a
50%	Hyssop	0.303 b	3.434 a	3.579 a	9.954 a	3.658 a	2.731 a	13.612 a	2.674 a
	Salvia	0.312 b	2.219 b	2.646 b	6.182 b	2.582 b	2.400 ab	8.764 b	2.138 a
	Oregano	0.441 a	3.360 a	1.454 c	5.123 b	2.566 b	1.997 b	7.688 b	2.553 a
75%	Hyssop	0.451 b	3.956 a	6.421 a	8.133 a	3.063 a	2.667 a	11.196 a	3.126 a
	Salvia	0.402 b	2.494 b	4.986 b	6.021 b	2.343 b	2.604 a	8.364 b	2.307 b
	Oregano	0.860 a	4.169 a	2.488 c	4.465 c	2.478 b	1.804 b	6.944 b	3.275 a
**Significance**	S	*	*	*	*	*	*	*	*
	D	*	*	*	*	*	ns	*	*
	S*D	*	*	*	*	*	*	*	ns

Data was evaluated via two-way ANOVA, followed by Tukey HSD test (mean, n = 4). Identical letters indicate that values do not differ significantly. Asterisks indicate significantly influential factors. MDA, malondialdehyde; Pro, proline; Chl a, chlorophyll *a*; Chl b, chlorophyll *b;* Total Chl, total chlorophylls; Chl a/b, chlorophyll *a/b* ratio; Car, carotenoids

Table S2-C: Results of 2-factorial ANOVA for non-enzymatic antioxidants.

Level of drought	Species	TPC	GSH	GSSG	GSH/GSSG	GSH+GSSG	AsA	DHA	AsA/DHA	AsA+DHA
25%	Hyssop	57.311 a	254.011 a	42.289 a	6.033 a	296.300 a	15.320 a	6.718 a	2.296 b	22.038 a
	Salvia	14.803 b	192.886 b	36.388 a	5.319 b	229.273 b	6.740 b	3.536 b	1.918 b	10.275 b
	Oregano	61.800 a	86.741 c	14.535 b	6.058 a	101.276 c	18.765 a	5.676 a	3.297 a	24.441 a
50%	Hyssop	71.040 b	233.768 a	50.107 a	4.656 a	283.875 a	25.166 a	11.220 a	2.410 b	36.386 a
	Salvia	21.584 c	194.674 b	44.022 a	4.441 a	238.696 b	12.229 b	5.995 b	2.051 b	18.224 c
	Oregano	83.911 a	77.422 c	22.200 b	3.502 b	99.622 c	24.310 a	6.315 b	3.841 a	30.625 b
75%	Hyssop	44.726 b	198.108 a	67.627 a	2.968 a	265.734 a	16.475 b	11.344 a	1.460 b	27.819 b
	Salvia	36.329 b	160.651 b	59.772 a	2.743 a	220.423 b	8.308 c	7.496 b	1.115 b	15.804 c
	Oregano	92.472 a	67.415 c	24.872 b	2.765 a	92.287 c	30.250 a	6.755 b	4.516 a	37.005 a
**Significance**	S	*	*	*	ns	*	*	*	*	*
	D	*	*	*	*	ns	*	*	ns	*
	S*D	*	ns	*	ns	ns	*	*	*	*

Data was evaluated via two-way ANOVA, followed by Tukey HSD test (mean, n = 4). Identical letters indicate that values do not differ significantly. Asterisks indicate significantly influential factors. TPC, total phenolic compounds; DW, dry weight; GSH, reduced glutathione; GSSG, oxidized glutathione; AsA, ascorbic acid; DHA, dehydroascorbic acid

Table S2-D: Results of 2-factorial ANOVA for antioxidant enzymes.

Level of drought	Species	SOD	CAT	APX	GPX	POD
25%	Hyssop	1.838 a	614.079 a	8.082 a	0.0830 b	0.831 b
	Salvia	2.096 a	378.384 b	8.682 a	0.482 a	2.705 a
	Oregano	0.885 b	121.444 c	13.874 b	0.0711 b	1.093 b
50%	Hyssop	3.971 a	391.582 a	7.194 a	0.224 a	1.382 b
	Salvia	2.309 b	239.057 b	7.484 a	0.277 a	2.696 a
	Oregano	0.773 c	191.362 b	15.200 b	0.0844 b	1.332 b
75%	Hyssop	4.419 a	291.457 a	6.782 a	0.426 a	2.054 b
	Salvia	3.039 b	248.309 a	9.651 b	0.103 b	2.742 a
	Oregano	0.936 c	317.486 a	21.491 c	0.0510 b	1.532 c
**Significance**	S	*	*	*	*	*
	D	*	*	*	ns	*
	S*D	*	*	*	*	*

Data was evaluated via two-way ANOVA, followed by Tukey HSD test (mean, n = 4). Identical letters indicate that values do not differ significantly. Asterisks indicate significantly influential factors. SOD, superoxide dismutase; U, units; CAT, catalase; APX, ascorbate peroxidase; AsA, ascorbic acid; GPX, glutathione peroxidase; GSH, glutathione; POD, peroxidase

Se-treatment: Se

Table S2-E: Results of 2-factorial ANOVA for growth characteristics and selenium concentration.

Level of drought	Species	PH	FW	DW	RWC	Se
25%	Hyssop	23.603 c	5.766 b	0.646 c	80.201 b	9.530 a
	Salvia	42.859 b	8.579 a	0.962 b	77.547 c	2.927 b
	Oregano	54.407 a	8.870 a	1.755 a	88.128 a	3.361 b
50%	Hyssop	24.342 b	5.007 b	0.626 c	78.685 a	8.891 a
	Salvia	40.462 a	7.507 a	0.945 b	76.540 ab	2.942 b
	Oregano	43.185 a	8.523 a	1.799 a	75.840 b	3.219 b
75%	Hyssop	18.070 b	2.924 b	0.431 b	64.872 b	7.862 a
	Salvia	31.909 a	4.716 a	0.635 b	69.381 a	3.299 b
	Oregano	36.745 a	5.095 a	1.033 a	63.073 b	2.712 b
**Significance**	S	*	*	*	*	ns
	D	*	*	*	ns	*
	S*D	*	ns	*	*	*ns

Data was evaluated via two-way ANOVA, followed by Tukey HSD test (mean, n = 4). Identical letters indicate that values do not differ significantly. Asterisks indicate significantly influential factors. RWC, relative water content

Table S2-F: Results of 2-factorial ANOVA for oxidative stress parameters and photosynthetic pigments.

Level of drought	Species	MDA	H2O2	Pro	Chl a	Chl b	Chl a/b	Total Chl	Car
25%	Hyssop	0.251 a	2.875 a	2.744 a	12.920 a	5.830 a	2.221 a	18.749 a	2.869 a
	Salvia	0.302 a	1.249 c	2.220 ab	8.303 b	3.625 b	2.331 a	11.928 b	2.161 b
	Oregano	0.167 b	2.076 b	1.588 b	5.874 c	2.307 c	2.572 a	8.181 c	3.071 a
50%	Hyssop	0.244 b	3.329 a	5.181 a	12.251 a	4.325 a	2.841 a	16.576 a	2.537 b
	Salvia	0.352 a	2.101 b	3.830 b	7.548 b	3.197 b	2.362 a	10.745 b	2.020 b
	Oregano	0.260 b	2.627 b	2.399 c	5.374 c	2.148 c	2.511 a	7.523 c	3.231 a
75%	Hyssop	0.392 a	3.640 a	6.559 a	9.383 a	3.658 a	2.584 a	13.041 a	2.882 ab
	Salvia	0.462 a	3.180 ab	5.485 b	4.877 b	1.915 c	2.560 a	6.793 b	2.601 b
	Oregano	0.567 a	3.048 b	3.258 c	5.256 b	2.402 b	2.194 a	7.658 b	3.223 a
**Significance**	S	*	*	*	*	*	ns	*	ns
	D	*	*	*	*	*	ns	*	*
	S*D	*	*	*	*	*	*	*	ns

Data was evaluated via two-way ANOVA, followed by Tukey HSD test (mean, n = 4). Identical letters indicate that values do not differ significantly. Asterisks indicate significantly influential factors. MDA, malondialdehyde; Pro, proline; Chl a, chlorophyll *a*; Chl b, chlorophyll *b;* Total Chl, total chlorophylls; Chl a/b, chlorophyll *a/b* ratio; Car, carotenoids

Table S2-G: Results of 2-factorial ANOVA for non-enzymatic antioxidants.

Level of drought	Species	TPC	GSH	GSSG	GSH/GSSG	GSH+GSSG	AsA	DHA	AsA/DHA	AsA+DHA
25%	Hyssop	94.728 a	273.213 a	41.252 a	6.641 a	314.464 a	21.280 a	6.344 a	3.432 ab	27.624 a
	Salvia	22.468 b	207.957 b	38.177 a	5.459 b	246.134 b	9.595 b	3.333 b	2.939 b	12.927 b
	Oregano	95.251 a	76.054 c	13.668 b	5.659 ab	89.721 c	24.513 a	6.155 a	3.985 a	30.668 a
50%	Hyssop	89.675 a	245.858 a	48.301 a	5.130 a	294.159 a	23.342 b	9.500 a	2.473 b	32.842 a
	Salvia	29.183 b	185.750 b	39.886 b	4.709 ab	225.635 b	12.454 c	6.468 b	1.940 b	18.922 b
	Oregano	101.721 a	78.538 c	20.885 c	3.833 b	99.423 c	29.637 a	7.262 b	4.162 a	36.900 a
75%	Hyssop	62.976 b	208.874 a	60.065 a	3.483 a	268.939 a	14.336 b	9.854 a	1.477 b	24.190 b
	Salvia	26.240 c	148.925 b	64.240 a	2.320 b	213.165 b	6.455 c	9.843 a	0.660 b	16.298 c
	Oregano	136.721 a	70.490 c	23.743 b	3.001 ab	94.233 c	33.955 a	7.991 b	4.288 a	41.946 a
**Significance**	S	ns	*	*	*	*	*	*	*	*
	D	*	*	*	*	*	*	*	*	*
	S*D	*	*	*	ns	ns	*	*	*	*

Data was evaluated via two--way ANOVA, followed by Tukey HSD test (mean, n = 4). Identical letters indicate that values do not differ significantly. Asterisks indicate significantly influential factors. TPC, total phenolic compounds; DW, dry weight; GSH, reduced glutathione; GSSG, oxidized glutathione; AsA, ascorbic acid; DHA, dehydroascorbic acid

Table S2-H: Results of 2-factorial ANOVA for antioxidant enzymes.

Level of drought	Species	SOD	CAT	APX	GPX	POD
25%	Hyssop	1.690 a	636.059 a	8.415 b	0.144 b	1.092 b
	Salvia	2.177 a	359.640 b	6.345 b	0.695 a	2.742 a
	Oregano	1.131 b	114.023 c	15.579 a	0.131 b	0.723 b
50%	Hyssop	2.836 a	507.244 a	8.346 b	0.297 b	1.233 b
	Salvia	2.309 b	266.090 b	10.089 b	0.398 a	2.858 a
	Oregano	1.001 c	303.631 b	22.239 a	0.153 c	1.037 b
75%	Hyssop	4.655 a	319.333 c	7.057 c	0.547 a	1.662 b
	Salvia	2.565 b	219.633 b	11.337 b	0.191 b	3.996 a
	Oregano	1.419 c	386.620 a	28.776 a	0.122 c	1.412 b
**Significance**	S	*	*	*	*	*
	D	*	*	*	*	*
	S*D	*	*	*	*	*

Data was evaluated via two-way ANOVA, followed by Tukey HSD test (mean, n = 4). Identical letters indicate that values do not differ significantly. Asterisks indicate significantly influential factors. SOD, superoxide dismutase; U, units; CAT, catalase; APX, ascorbate peroxidase; AsA, ascorbic acid; GPX, glutathione peroxidase; GSH, glutathione; POD, peroxidase
